# Road crossings change functional diversity and trait composition of stream-dwelling macroinvertebrate assemblages

**DOI:** 10.1038/s41598-023-47975-z

**Published:** 2023-11-24

**Authors:** Blanka Gál, András Weiperth, János Farkas, Dénes Schmera

**Affiliations:** 1 Balaton Limnological Research Institute, Klebelsberg K. u. 3, 8237 Tihany, Hungary; 2 National Laboratory for Water Science and Water Security, Balaton Limnological Research Institute, Klebelsberg K. u. 3, 8237 Tihany, Hungary; 3 Department of Freshwater Fish Ecology, Institute of Aquaculture and Environmental Safety, Hungarian University of Agriculture and Life Sciences, Páter Károly u. 1, Gödöllő, 2103 Hungary; 4 Department of Systematic Zoology and Ecology, Institute of Biology, ELTE Eötvös Loránd University, Pázmány Péter sétány 1/C, 1117 Budapest, Hungary

**Keywords:** Ecology, Biodiversity, Community ecology, Freshwater ecology, Urban ecology

## Abstract

Functional diversity is regarded as a key concept in understanding the link between ecosystem function and biodiversity, and is therefore widely investigated in relation to human-induced impacts. However, information on how the intersection of roads and streams (hereafter road crossings, representing a widespread habitat transformation in relation to human development), influences the functional diversity of stream-dwelling macroinvertebrates is still missing. The general aim of our study was to provide a comprehensible picture on the impacts of road crossing structures on multiple facets of the functional diversity of stream-dwelling macroinvertebrates. In addition, we also investigated changes in trait structure. Our research showed that road crossing structures had negative impacts on functional richness and dispersion; i.e., functional diversification. However, we found no significant impact on functional divergence and evenness components. We found a decrease in functional redundancy at road crossing structures. This indicates a reduced ability of the community to recover from disturbances. Finally, we found that road crossings drive stream habitat and hydrological changes in parallel with modification of the trait composition of stream-dwelling macroinvertebrate assemblages. All these results suggest that road crossings cause notable changes in the functional diversity of stream-dwelling macroinvertebrate assemblages.

## Introduction

Human populations have been transforming the environment for millennia. This process influences the environment at the global scale since transformation extends in space and increases in intensity. Similarly, freshwater ecosystems have been transforming globally. For example, 77% of rivers no longer flow freely from the source to the sea because of riparian fragmentation and flow regulation^1^, while global trends show a decline in freshwater quality from 1970 to the present^2^. Unfortunately, human-induced impacts are often associated with a decline in biodiversity^3^. Consequently, the freshwater ecosystem’s capacity to provide critical ecosystem services has also declined, including environmental processes that support human health and quality of life^2^. The expansion of roads is a major factor in triggering land use change and also poses a significant threat to biodiversity per se^4^. Humans are rapidly approaching the point where they disintegrate the remains of biologically rich and environmentally important ecosystems with roads^5,6^. The expansion of roads is estimated to increase globally by 25 million kilometres by the year 2050^7^.

Along with other human developments, roads and road crossings (bridges and culverts) have major impacts on freshwater habitats and ecosystems^8^, as well as transforming ecological and hydrological connectivity. Road crossings change the physical structure of channel morphology and the substrate composition of a riverbed, therefore altering hydrology and reducing freshwater habitat quality and heterogeneity^9–11^. Road crossings often have their own drainage systems that transport road runoff directly into streams. Runoff may bring sediment from erosion or mass wasting during rainfall events and can degrade freshwater habitats^12–14^. Runoff may contain heavy metals, pesticides, de-icing salt and organic pollutants^15–18^. Runoff may transfer polymer components, especially minute particles from car tires^19^, which are estimated as one of the largest sources of microplastics in several countries^20–22^. Consequently road crossings likely represent an important pathway from land to freshwater systems for the release of microplastic particles^19^.

Road crossings affect freshwater ecosystems through multiple pathways. However, there is still a lack of information about the underlying threat to freshwater ecosystems, especially freshwater macroinvertebrates. The few studies investigating the impacts of road crossings on macroinvertebrate assemblages found species loss and altered community composition^11,23,24^. These findings consider taxa as being equally distinct from one another, and disregard the fact that communities are composed of species with a diverse array of ecological functions^25^. To address this issue, the last decade has seen a growing interest in alternative representations of biodiversity including functional diversity. Functional diversity quantifies the components of biodiversity that influences how an ecosystem operates or functions^26^.

Taxonomic and functional diversity can provide complementary information^27,28^ however, there is evidence suggesting that patterns in taxonomic diversity do not necessarily reflect changes in functional diversity^29,30^. Studies on functional diversity revealed that functional homogenization can be greater as expected from taxonomic dissimilarity^29^. Moreover, functional diversity is a multifaceted concept that can be characterized by a limited number of primary components such as functional richness, evenness, divergence, dispersion^31,32^, and can be characterized by functional uniqueness and redundancy^33^. Functional diversity components are complementary and together they can describe the distribution of species and their abundances within functional space^34^. Since each component describes an independent aspect of functional diversity^35^, it is beneficial to measure the different components to clarify a complete quantification of functional diversity^34^.

Functional richness is a measure of the overall trait space occupied by an ecological community^32^ and relative to the intensity of land use change, it can decline without concomitant changes in species richness^36^; i.e., it is independent from the rate of loss in taxonomic richness^37^. We hypothesized that anthropogenic disturbance may change environmental factors that can remove some species with an extreme combination of trait states, which occur on the edge of trait space^32^, and thus functional richness will decrease. As taxonomic diversity declines near road crossings^11^, our hypothesis suggests that functional richness and taxonomic diversity show a similar response to disturbance. Moreover, we assumed that disturbance can change the regularity of the distribution and the relative abundance of species in a community, which can affect functional evenness^31,38^. We therefore hypothesized that disturbances near road crossings reduce or completely eliminate the abundance of some combinations of trait states (e.g. univoltine taxa which are rare and respire through gills see: Barnum, et al.^37^) and thus functional evenness will decrease. Another functional diversity component is functional divergence that is related to the value of species abundances present at the edge of trait space^39^. We hypothesized that functional divergence would change at road crossings since environmental stress may redistribute community patterns in trait space. It can both increase (in the direction toward the fringe) or decrease (by eliminating species with some combination of trait states)^37^. As such, functional divergence may indicate a particular pattern of community functional composition^40^. Conversely, a functional dispersion index has the benefit of not being influenced by taxonomic richness, and measures the dispersion of species in trait space from the centroid. This index can be unweighted or weighted by the relative abundance of different taxa^39^, and gives higher values when there are taxa with traits differing extremely from mean community trait values. Road crossings can affect the abiotic environment through multiple pathways which can act as trait filters, thus it can create relatively limited functional differences among species. Consequently, we hypothesized that functional trait differences (functional dispersion) will decrease at road crossings.

Anthropogenic disturbances can cause loss of species and consequently ecological functions. Functional redundancy exists when the species within a community share all biological characteristics and thus perform similar ecosystem functions. On the other hand, functional uniqueness refers to the situation when species do not share any biological characteristics^33^. From a disturbance perspective, a community which has functionally redundant species could go locally extinct without considerable loss in ecosystem function^41^. Therefore functional overlap supports ecosystem stability and acts as a resilience to loss in community function^42^. Disturbances can eliminate unique combinations of trait states from the community, consequently increasing functional redundancy and decreasing uniqueness. We therefore hypothesized that functional originality (redundancy and uniqueness) will change as a result of disturbances.

Finally, we hypothesized that road crossings will influence the functional composition of communities. We predicted that road crossings change the environment, and this change will be reflected by traits-based community structure.

## Results

**Figure 1:**
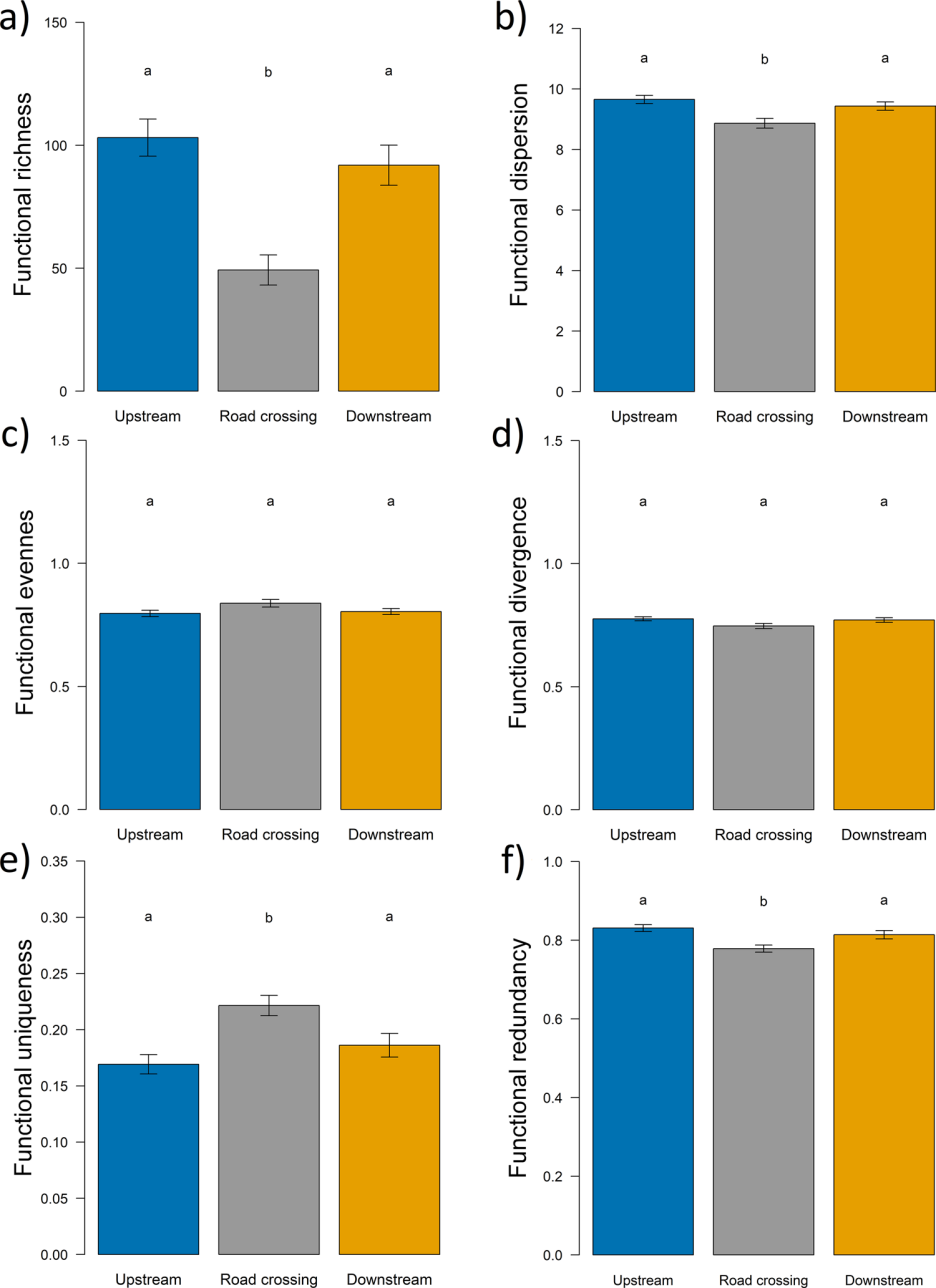
Effect of stream section (upstream [blue], road crossing [grey] and downstream [yellow]) on (**a**) functional richness, (**b**) functional dispersion, (**c**) functional evenness, (**d**) functional divergence, (**e**) functional uniqueness, (**f**) functional redundancy of macroinvertebrate taxa. Bars show mean values, whiskers standard errors. Different letters indicate significant differences by Tukey test.

The best fit model (with the lowest AICc) identified that the functional richness of the macroinvertebrate community was influenced by stream section, site and season (Table 1). Alternative statistical models were not plausible (Table 1). Tukey tests showed that road crossings had a negative effect on functional richness; thus upstream sections had the highest functional richness followed by downstream and road crossing sections (Fig. 1a).

**Table 1:** Best fit linear models explaining the effects of stream section, site and season on the on functional richness, functional dispersion, functional evenness and functional divergence.

Response variable	Predictors	df	AICc	Delta AICc	Weight
Functional richness	Stream section + season + site	14	1881.2	0.00	0.668
	Stream section + site	12	1882.6	1.40	0.332
Functional dispersion	Stream section + season + site	14	497.9	0.00	0.948
	Stream section + site	12	503.7	5.86	0.051
Functional evenness	Stream section + season + site	14	− 311.4	0.00	0.357
	Season + site	12	− 311.2	0.18	0.326
	Stream section + season	6	− 310.0	1.36	0.181
	Season	4	− 309.5	1.94	0.135
Functional divergence	Stream section + site	12	− 440.1	0.00	0.519
	Site	10	− 439.5	0.62	0.382
	Stream section + season + site	14	− 435.6	4.48	0.055
	Season + site	12	− 435.0	5.06	0.041

Only models with delta AICc < 10 are displayed.

Concerning functional dispersion, the best fit model revealed an effect of stream section, site and season (Table 1). Alternative statistical models explaining functional dispersion of the macroinvertebrate communities were not plausible (Table 1). The Tukey test showed that upstream sections had higher functional dispersion than road crossings and downstream sections (Fig. 1b).

There were four plausible statistical models of functional evenness (Table 1). Our information theoretic approach revealed that the best models explained the importance of a significant effect of seasons (three models), while two models demonstrated the importance of stream section and sites. The Tukey test showed that road crossings had the highest functional evenness followed by downstream and upstream sections (Fig. 1c).

There were four plausible statistical models of functional divergence. Most models suggested the importance of site (four models) whereas two models showed the importance of stream section and season (Table 1). A Tukey test showed that road crossing had the lowest functional divergence followed by downstream and upstream sections (Fig. 1d). The ANOVA table of the best fit model showed that stream section had a significant effect on the functional uniqueness and redundancy (Table 2). The Tukey test showed that road crossing had the highest functional uniqueness followed by downstream and upstream sections (Fig. 1e). On the contrary, redundancy showed that upstream section had the highest functional redundancy followed by downstream and road crossing (Fig. 1f).

**Table 2:** Summary output of ANOVA table explaining the effect of stream section on functional uniqueness and redundancy of macroinvertebrate communities.

Predictor	Df	Sum Sq	Mean Sq	F value	P
Stream section	2	0.074	0.037	6.931	0.001
Residuals	172	0.921	0.005		

**Figure 2:**
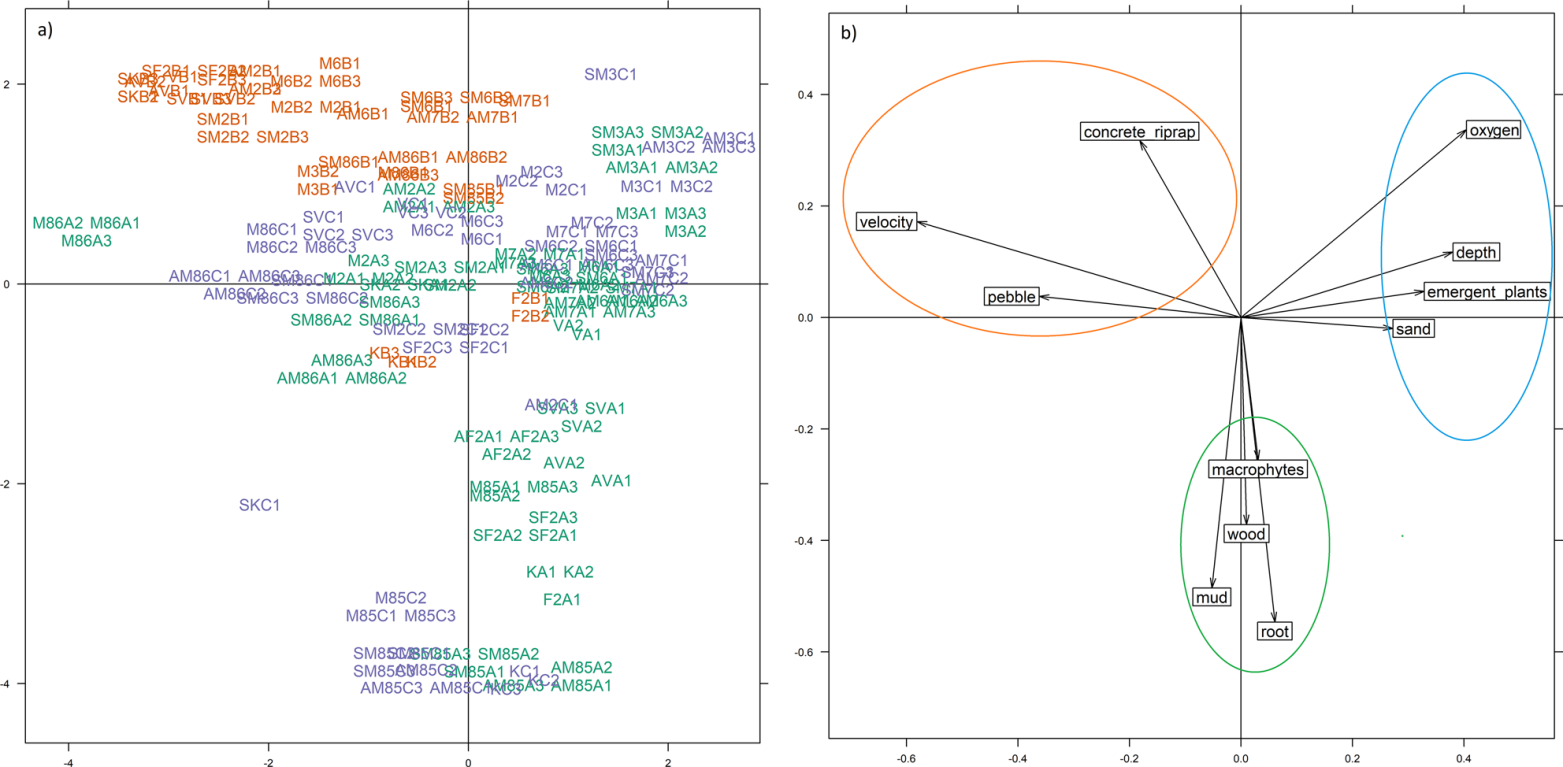
Result of the first two axes of RLQ analysis: (**a**) the different sampling sites positioned by their environmental conditions (green: upstream, brown: road crossings, purple: downstream, (**b**) environmental variable scores (orange: first habitat type, blue: second habitat type, green: third habitat type).

Results of the RLQ permutation tests (n = 9999) revealed that overall, the different environmental variables influenced the distribution of macroinvertebrate species (model 2, P = 0.0001) although traits did not influence the composition of species under certain environmental conditions (model 4, P = 0.2605). Individual traits still changed as a result of the different environmental variables at stream section (see below for the result of the fourth-corner analysis). The first two RLQ axes explained 85.15% of the total variance (first axis: 50.64%; second axis: 34.50%) across functional traits and environmental variables (Table 3). The first axis accounted for 77% of the variability of the stream sections and 70% of the variance of the trait table (Table 3). The RLQ analysis revealed that road crossing sections clearly separated from the upstream and downstream sections according to the environmental variables (Fig. 2), while a clear distinction was not evident between upstream and downstream sections. According to the environmental variables we can distinguish three main types of habitats (Fig. 2b). The RLQ analysis also highlighted relationships between the traits and the taxa (Suppl. Figure 2).

**Table 3:** Summary of the RLQ analysis.

RLQ analysis	Axis 1	Axis 2
Eigenvalue	5.389	3.671
% of total co-inertia	50.6%	34.5%
Covariance	2.321	1.916
Correlation	0.561	0.482
Cumulative inertia (environment)	2.329	4.963
Ratio (environment)	77%	90%
Cumulative inertia (traits)	7.337	13.323
Ratio (traits)	70%	71%

The fourth-corner analysis extracted a total of 49 significant bivariate trait-site relationships (Supp. Figure 3). The traits within all grouping features (11) showed significant bivariate association with all the environmental variables. According to the feeding habits shredders were negatively associated with velocity and positively associated with emergent terrestrial plants, emergent macrophytes and higher dissolved oxygen. Filter-feeders were positively associated with concrete and riprap, while (animals and plant) piercers were associated with wood and mud. Aquatic invertebrates inhabiting interstitial spaces were negatively associated with dead wood and branches, roots of terrestrial plants and mud. Macroinvertebrates that are temporarily attached to hard substrates were positively associated with velocity and concrete or riprap substrate, whereas these taxa were negatively correlated with sand. Organisms using gills for respiration were negatively associated with dead wood and branches and mud. Aquatic passive dispersers were positively correlated with concrete/riprap and were negatively associated with floating and submerged macrophytes, dead wood and branches, roots of the terrestrial plants and mud. Aerial passive and active dispersers were positively associated with current velocity and negatively associated with depth, emergent plants and sand. Small macroinvertebrates (length is 0.25 cm or less) were positively associated with habitats characterized by mud with dead wood and branches. Deeper water levels were associated positively with macroinvertebrates with a life duration lasting > 1 year. Organisms that spend their life in aquatic systems as nymph or pupa were positively associated with velocity and concrete/riprap streambed and negatively associated with sand. Macroinvertebrates that spend their life in aquatic systems as adults were positively associated with water depth while organisms that have only one generation per year (monovoltine species) were negatively associated with water depth. Ovoviviparity was negatively associated with velocity and pebbles, and positively associated with emergent vegetation, oxygen and sand. Macroinvertebrates that reproduction with free- isolated eggs were positively associated with macrophytes. Cemented or a fixed-clutch reproduction type was positively associated with velocity and negatively associated with emergent plants and sand. Macroinvertebrates that shred dead vegetation were positively associated with water depth and negatively associated with mud. Invertebrate species that withstand unfavourable conditions as eggs were positively associated with velocity and pebbles, and negatively associated with emergent plants.

**Figure 3:**
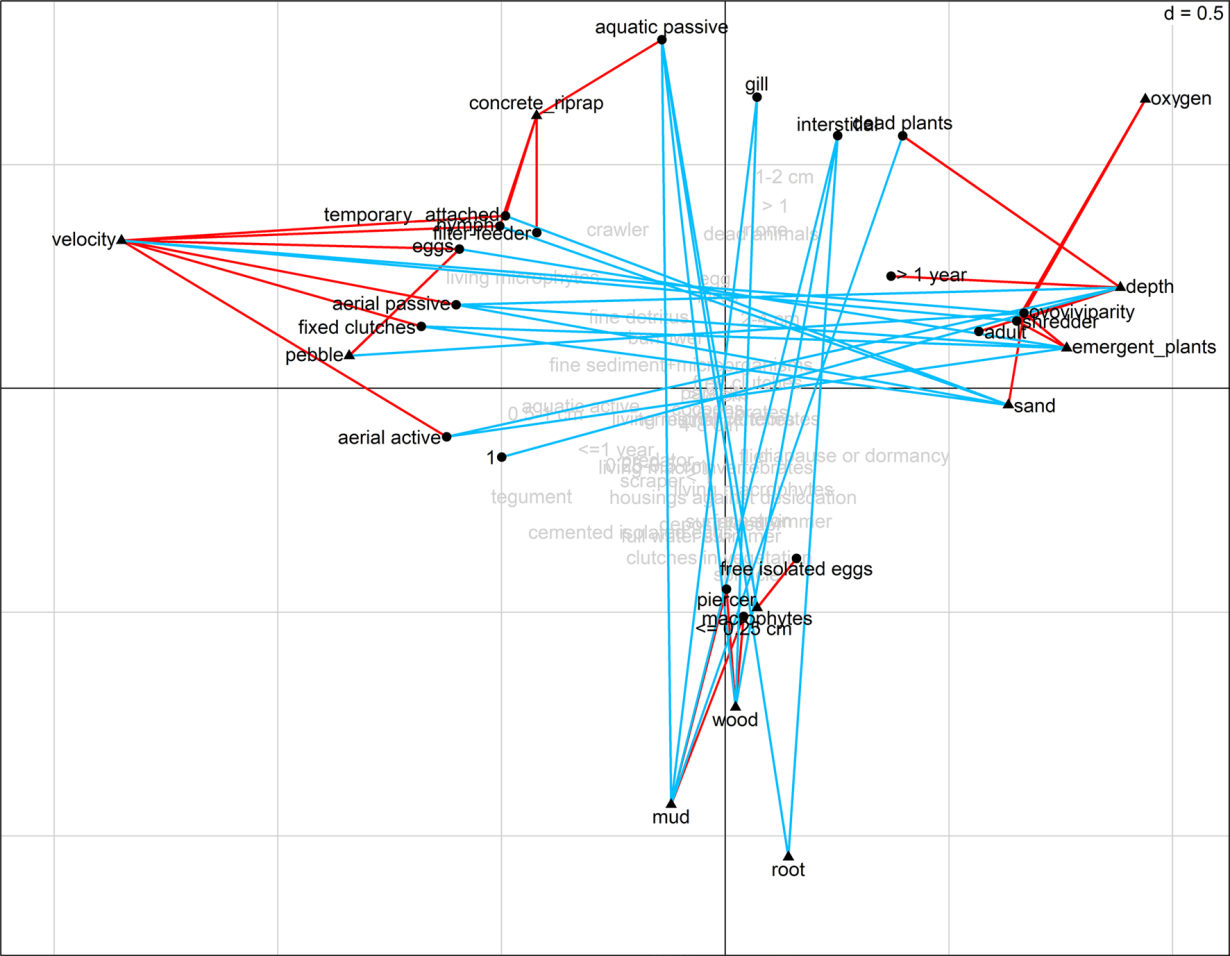
Biplot of the combination of RLQ ordination and fourth-corner analysis. Significant (p < 0.05) positive associations are represented by red lines and significant negative association by blue lines. Variables and traits with no associations are displayed in light grey. Environmental variables are triangles, while functional traites are represented by circles.

Combined fourth- corner and RLQ analysis revealed that the environmental variables formed three main type clusters; i.e., three main habitat types (see Figs. 2b and 3): (1) the first habitat type had generally higher flow velocity, and the dominant substrates were pebbles and artificial riprap or concrete, and were negatively correlated with the first axis. (2) the second habitat type contained two other main habitat types, related to the natural upstream and downstream sections. One of the clusters was associated with deeper sites and characterized by sand, higher dissolved oxygen concentration, emergent terrestrial plants and emergent macrophytes (*Typha* sp. Linnaeus (1753), *Carex* sp. L., *Phragmites* sp. Adans.) which were positively correlated with the first axis. (3) The last habitat type was characterized by a high proportion of floating and submerging macrophytes, mud, dead wood and branches and fine roots and negatively correlated with the second axis.

**Figure 4:**
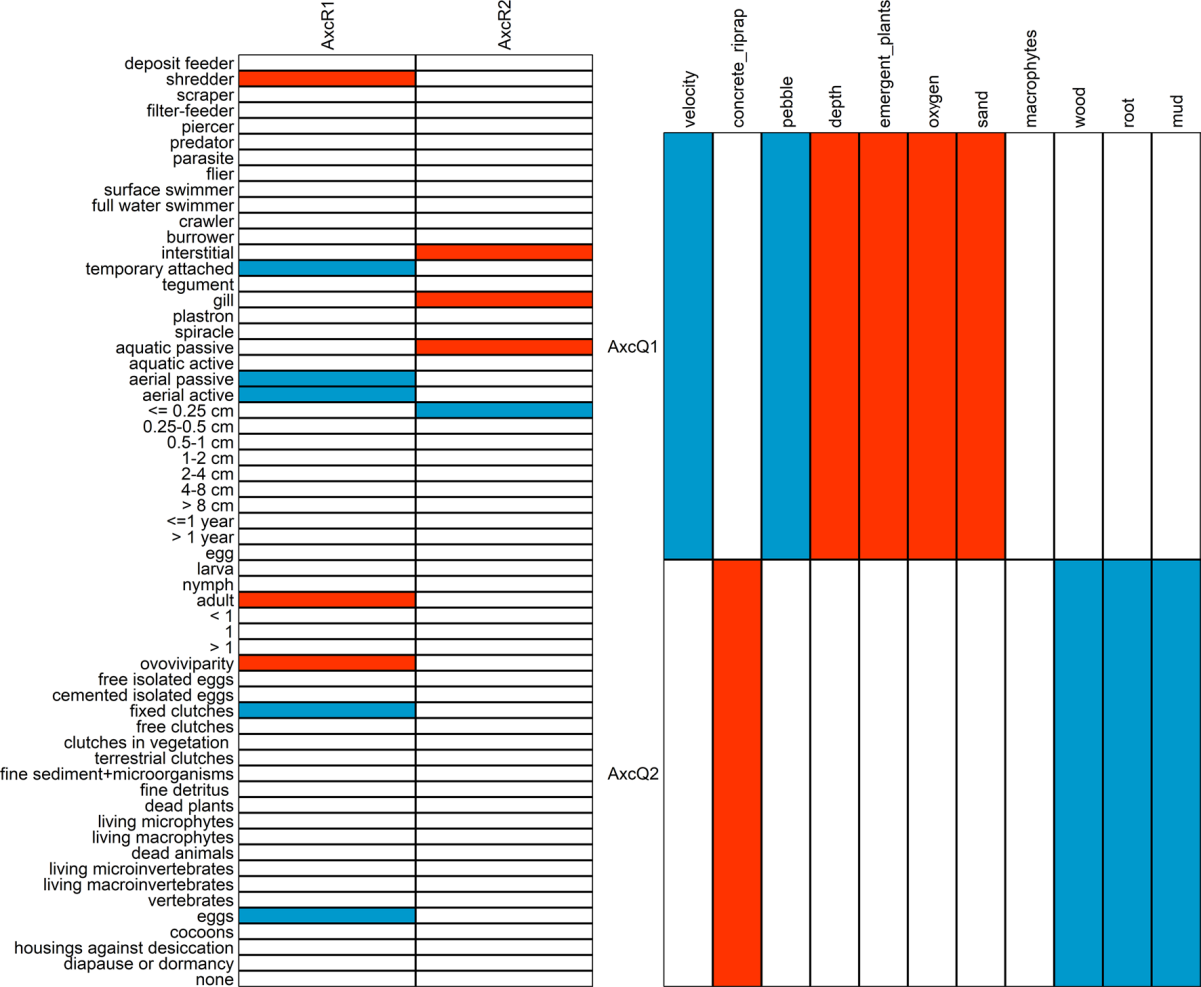
Significant correlation between (**a**) functional traits and RLQ ordination axes and (**b**) between environmental variables and RLQ ordination axes. Red = positive correlations, blue = negative correlations and white = no correlations.

As for the RLQ ordination (Fig. 3), filter- feeders, aquatic passive dispersers, aerial passive and active dispersers, macroinvertebrates that were temporarily attached to hard substrates, and spend their life in aquatic systems as nymph or pupa, reproduction with cemented or fixed clutches and survivor unfavourable conditions with eggs grouped together. Conversely, macroinvertebrates that are shredders, life duration > 1 year, spend their life in aquatic systems as adults, are ovoviviparous and feed on dead plant matter (≥ 1 mm) were all positively correlated. Finally, macroinvertebrates that are small (length is 0.25 cm or less), piercers, and oviposit single eggs in the water freely form a group together. These results show that the three clusters of functional traits overlap with the clusters of the environmental variables. These patterns were reinforced by the strength and significance of each environmental variable and functional traits against the appropriate RLQ axes. (AxQ1, AxQ2, AxcR1, AxcR2) (Fig. 4).

## Discussion

Functional assessment of ecological communities is receiving increasing attention. Here we examined the impacts of road crossing structures on the functional diversity and trait composition of stream-dwelling macroinvertebrates. We found that road crossing structures had negative impacts on functional richness and dispersion, both quantifying functional diversification of the communities. We observed that road crossings had no impact on functional divergence and evenness but increased functional uniqueness and decreased functional redundancy. Finally, traits-based analyses showed that road crossings influence community structure. All of these results suggest that road crossings structure several functional aspects of stream-dwelling macroinvertebrate assemblages.

In accordance with our hypothesis, we found that road crossings had a negative effect on functional richness. Studies examining the relative role of environmental gradients in shaping trait composition revealed that variation in functional richness can be explained by local environmental variables^43,44^. Anthropogenic disturbances such as flow regime alteration, impervious surface coverage and agricultural intensity can lead to changes in local environmental conditions, and these changes can reduce functional richness^37,45–47^. Road crossings generally have concrete or riprap stream bed, and these artificial structures can reduce habitat heterogeneity^11^ which may have effect on the functional richness. Our results concur with these findings because at road crossings, the environmental variables were typically different from those present under natural conditions. Moreover, road surface run-off may contain different chemicals^18^. These environmental filters can eliminate some species with extreme trait values and reduce the extension of the functional trait-space filled by the community. We observed that not only the stream section (upstream, road crossing, downstream), but also site identity influenced functional richness. In other words, the different study sites differed in terms of functional richness. This can be explained by the difference in the regional environment variables, which results in a contrasting regional species pool of study sites. As for the significant effects of season, it could be caused by natural flow regime, different water temperature between seasons and the natural seasonal life cycle of macroinvertebrates^48^. Our finding that functional richness decreased at road crossings (present study) together with the finding that taxonomic richness decreased also with stream crossings^11^ suggest that taxonomic richness is associated with higher functional richness. Empirical studies such as Heino^27^, Leigh et al.^28^, Bêche et al.^49^, Larsen and Ormerod^50^, Ding et al.^51^ confirm this conclusion.

As expected, functional dispersion was lower at road crossing sections compared to the natural sections. This finding could be because road crossings can affect the abiotic environment through multiple pathways, thus relatively few species with only specific combinations of trait-states can overcome these complex filters^52^. This is reflected in decreased trait diversity and greater dominance of fewer and similar traits. In most cases, disturbed streams were dominated by small-bodied, short-lived and highly reproductive organisms with more generalist feeding traits e.g. collector-gatherers organism, like Chironomidae larvae^50,53^. We also observed significant effects of stream section as well as that of season and site on functional dispersion. This result implies that functional dispersion varied across habitats and seasons. The high spatial–temporal variability could be relevant for communities characterized by short-lived and mobile species where trait composition can shift quickly^54,55^. We also found a positive relationship between functional dispersion and functional richness^39^.

However, functional richness and dispersion showed a significant change at road crossings, with functional evenness and functional divergence components being less sensitive to disturbance. Contrary to our study, Barnum et al.^37^ reported decreasing functional evenness with increasing impervious surface coverage; and Gerisch et al.^56^ also found that functional evenness decreased with increasing disturbance. We found a negligible increase in functional evenness near road crossings. This result indicates there was a slightly more homogenous distribution of functional traits in the niche space, and might reflect more intensive resource utilization^31^, which may have been mainly due to reductions in available resources at disturbed sites. High functional divergence suggests a high degree of niche differentiation and thus low resource competition^31^, which would be reasonable since we assumed there were lower available resources at road crossings. However, functional divergence was predicted to change (increase or decrease) with disturbance, and we did not find any differences. The similar functional evenness and divergence implies that the distribution of the abundance of functional traits in the niche space was broadly uniform at road crossings and natural sites.

As expected, there was a difference in the functional redundancy and uniqueness of macroinvertebrate assemblages between road crossings and natural sites. Functional uniqueness increased while functional redundancy declined in response to road crossings. Our findings therefore contrast with previous studies, which suggests that disturbances homogenize assemblages through the elimination of functionally unique species while increase redundant ones^36,46,57^. However, if there are interspecific interactions within a community such as competition for limited resources, it could limit the functional similarity between species^58,59^. Our result suggests that, despite road crossings being characterized by species with similar traits (due to decreased functional richness and dispersion), there are only a few overlaps between them. Thus, most of the functional trait combinations are represented by single species near road crossings. Therefore, we assume that macroinvertebrate species with unique trait combinations appeared to benefit from disturbance and fully exploit available resources. Conversely, communities with several functional redundant species show resilience and are stable against disturbances^47^, because the function of eliminated species is replaced by functionally identical species^42,60,61^.

The fourth-corner and RLQ analyses confirmed that the distribution of traits was not random, and environmental variables explained differences in the trait composition of macroinvertebrates collected in different stream sections. These results support the hypothesis that road crossings caused change in environmental variables, and these variables can filter traits of species within the community. The combined fourth-corner and RLQ analyses allowed us to infer the covariation, as well as the co-structure between the traits and environmental variables. The analysis revealed that the environmental variables formed three main types of clusters, hereafter named habitat types. The first habitat type complied with the road crossing sections (see Fig. 2a,b), while the other two types both with the upstream and downstream sections. Therefore, we conclude that road crossing sections had homogenous environmental variables, whereas the environmental variables of upstream and downstream sections were characteristic of typical lowland streams in Hungary. These habitats overlapped with the three clusters of functional traits (see Fig. 3).

The first habitat type was typical of the stream bed at road crossing structures which were defined by higher flow velocity and the substrates were mostly pebbles and artificial riprap or concrete. At road crossings, artificial streambeds are generally undersized. Therefore, the width of the stream at road crossings is narrower than at the upstream section. At high water levels, the narrowing increases flow velocity^62^. Moreover, road crossings often have their own drainage systems that transport road runoff directly into streams resulting in flashy hydrology^8^. Increased flow was one of the most influential roles in shaping the trait composition of macroinvertebrates near road crossings. In particular, the increased flow velocity supported the transport of fine organic material within the water column and therefore favoured the prevalence of filter- feeders^63–65^. In addition, some types of substrates can act as refugia, providing shelter against high flow velocity that may otherwise cause dislodgement. Theodoropoulos et al.^65^ found that boulder and large stones host higher macroinvertebrate abundance compared to finer substrates after a high flow event. Furthermore, they found increased relative abundance of filter- feeders in this flow refugia. The artificial stream bed near road crossings was often riprap which can act for filter-feeders such as *Hydropsychidae* and *Mysidae* species in the same way. At a few sites, there was pebble among pieces of boulders and at stream margins the bottom was covered by sand. This substrate was suitable for soft substrate-dweller filter- feeders such as *Pisidium* and *Sphaerium* species. We found a positive association between artificial stream beds with high flow velocity and macroinvertebrates with adaptations such as hooks, suction structures, or fixed cases for attachment to the substrate. These features are an adaptation for invertebrates to overcome fast flow, and therefore these temporary attachments reduce the likelihood of passive drift (i.e. Ephemroptera and Trichoptera species)^66^. In addition, we found that flow can influence the reproduction type of the macroinvertebrates. Thus, eggs had also increased attachment mechanisms such as cemented or fixed clutches to prevent drift dispersal^52^. Macroinvertebrates near road crossings were assigned into three dispersal modes: areal active, areal passive and aquatic passive. Areal active dispersers, such as Ephemeroptera and Trichoptera species, have terrestrial winged adult with an active dispersal mode that enables regular recolonization of disturbed habitats, whereas areal passive dispersers are usually small organisms which can be passively dispersed by wind, large-sized flying insects or birds. Conversely, lot of aquatic insects near road crossing structures have a tendency to utilize the unique prospect given by the higher flow velocity, thus disperse passively i.e., drifting. Drift could be a response to some stimulus like reduced food resources^67^, or anthropogenic or natural disturbance^68^. On the other hand, drift is regarded as an important mechanism for recolonizing reaches impacted by anthropogenic or natural disturbance^69^ from the upstream regions. In our case, taxa from various taxonomical groups such as *Baetis* Leach, 1815 larvae, *Hydropsyche* Pictet, 1834 larvae, *Limnomysis* Czerniavsky, 1882, *Dikerogammarus* Martynov, 1925, *Echinogammarus* Stebbing, 1899, *Pisidium* C. Pfeiffer, 1821, *Sphaerium* Scopoli, 1777 and *Potamopyrgus* Stimpson, 1865 use this passive dispersal strategy. We found that high velocity together with concrete or riprap streambed were positively associated with macroinvertebrates existing as nymph or pupa in aquatic systems during their life cycle i.e. with amphibiotic macroinvertebrates. This supported what we found with dispersal mode, because the adult form of amphibiotic macroinvertebrates is usually winged and dispersed aerial passive or active mode. The novel environmental conditions resulting from road crossings favoured invertebrate species that have evolved egg laying strategies to survive disturbances. These resistance forms allow organisms to recolonize sites after disturbance, thus supporting the organism’s resilience^70^. Specifically, adult female *Baetis* require emergent large rocks to crawl over underwater and attach their egg masses to the upper surface of rocks, whereas adult female *Hydropschidae* swim through the water column to oviposit underneath submerged substrates^71–73^. Road crossings often have their own stormwater-drainage systems which rapidly deliver stormwater from roads into streams, generating flashy hydrology^8,74^. Both taxa’s eggs can be vulnerable to flashy hydrography because partially submerged rocks at high water levels that are suitable for oviposition can become fully dry following peak flow, as experienced earlier due to water releases from hydropower dams^72^.

The upstream and downstream sections were differentiated into two habitat types. One habitat type was deeper and characterized by sand, higher dissolved oxygen concentration, emergent terrestrial plants and emergent macrophytes (*Typha*, *Carex*, *Phragmites*). Shredders were associated positively with these habitats because of the high proportion of organic matter accumulating between the stems of emergent plants. Shredders were represented mostly by amphipod crustaceans (*Gammarus*, *Echinogammarus*, *Dikerogammarus* sp.) and the isopod crustacean *Asellus aquaticus*. Besides, dead plant eater, long living (> 1 year) and ovoviviparous gastropods (*Potamopyrgus* and *Viviparus* sp.) dominated these sites. Ovoviviparity was associated with higher oxygen concentrations, contradicting previous studies that showed that ovoviviparity was less sensitive to oxygen depletion and may prevent high egg mortality under harsh environmental conditions (i.e., disturbed environments)^75,76^. However, Kuzmanovic et al.^77^ found that the presence of pesticides was associated with egg protection traits such as ovoviviparity, even under high oxygen content. Although our study did not consider pesticides or other chemical stressors, we assumed that some upstream and downstream sites received these chemicals due to neighbouring agricultural activity, which could contribute to the high number of ovoviviparous species. Moreover, a previous road crossing study found that downstream sections had more reduced diversity than upstream sections, and suggested that run-off at road crossings may have had negative impacts not just directly at the road crossings but also at downstream sections^11^. In addition, Murphy et al.^78^ found that fine organic sediment can increase the prevalence of ovoviviparity and adult aquatic stages in amphipods, consistent with our findings. Ovoviviparity can be advantageous in environments where eggs can be easily smothered by sediment, and adult aquatic stages also contribute to the resilience of organisms^76,78^.

The last habitat type, which characterised some upstream and downstream sections, was the most diverse with a high proportion of floating and submerging macrophytes, mud, dead wood and branches and living parts of terrestrial plants such as fine roots. Piercer macroinvertebrates, represented by a rich predatory Heteroptera fauna, had a positive association with mud, dead wood and branches at these sites. These predatory piercers inhabit mostly well-vegetated, stagnant or slow flowing waters^79,80^, however experimental studies found that dense vegetation and other physical structures that increase the complexity of the habitat affect the predation success of Heteropteran species^81,82^. While the movement of predators may be obstructed by habitat complexity, the presence of vegetation provides shelter for prey to evade predation^82,83^. Mud with dead wood and branches provide good perching sites for heteropteran species without impeding the movement of predators. We found that macroinvertebrates with a potential maximum size up to 0.25 cm were positively associated with this habitat. Other studies have reported an association between small body size and polluted stream sites^84^. This is consistent with the habitat template concept which predicts that large body sizes are often associated with fewer offspring per reproduction event due to a stable environment and short generation times; thus small organisms are associated with disturbed habitats^85^. However, we cannot say whether our result is consistent with the habitat template concept or not, because we had to exclude abundant and small sized road crossing related invertebrates such as *Chironomidae*, *Oligochaeta* and *Simuliidae* larvae from our analysis (see “Methods” section), which may affect our results. *Chironomidae* and *Oligochaeta* species are tolerant to a wide range of environmental conditions and disturbance^86^, while small filter- feeding *Simuliidae* larvae can be found near road crossings due to high flow velocity^87^. Without the exclusion, we might conclude that the small size macroinvertebrates are more likely associated with the disturbed road crossings than with these natural sections. Moreover, small bodied organisms were represented by Heteroptera species at this natural habitat, including *Micorvelia* species, *Plea minutissima* and *Micronecta scholzi*. Generally, Heteroptera species are considered to be weak biotic indicators because they have the ability to colonize a wide range of habitats regardless of the ecological conditions^88,89^. In addition, we found a positive association between macrophytes and macroinvertebrates reproducing with free eggs. The slow velocity, which can enhance the growth of dense macrophytes, tends to favour organisms that deposit eggs on the water surface without any protection, such as Ephemeroptera and Odonata species^72,90,91^. Macrophytes can reduce turbidity and stabilize sediments^92^ which can protect eggs from smothering, and under these conditions, species do not have to invest energy into parental care like ovoviviparity^76,78^.

Road crossings are complex habitats characterized by conditions that differ from natural conditions. Artificial substratum and flow velocity were the most important environmental factors shaping the trait composition of macroinvertebrates at road crossings. Flow velocity imposed different adaptation strategies such as hooks, suction structures, or induced eggs to develop mechanisms for attachment (cemented or fixed clutches). Artificial substratum, together with the impact of flow velocity, supports filter- feeders. Diverse active and passive dispersion abilities, and the production of eggs to survive harsh environments, are traits that can enhance the resilience of organisms under harsh environmental conditions. Overall, the significantly lower functional richness and functional dispersion values at road crossings indicated that the disturbance had strong negative effects on the functional diversity of the communities.

In conclusion, road crossings are an important environmental filter that can reduce environmental quality, while changing functional diversity and trait composition. As consumers at intermediate trophic levels, stream- dwelling macroinvertebrates in streams are influenced by bottom-up, as well as top-down, forces^93^. Thus, simplification of macroinvertebrate functional diversity may affect ecosystem functioning and services^94^, and modify species interactions, energy and material flows, and food-web stability^95,96^. In particular, functional redundancy also showed a significant decrease near road crossings, which could reduce the ability of the community to overcome new anthropogenic disturbances. The strong negative effects of road crossings on the functional diversity and functional redundancy of the macroinvertebrate assemblages emphasizes the importance of maintaining the local integrity of stream ecosystems. Installing properly sized road-crossing structures which can handle large storm flows and floods, can mitigate serious hydrological alteration. Ideally, streambed material at road crossings should be the same quality and size as the upstream and downstream sections, thus supporting stream habitat heterogeneity and preventing habitat alteration. Considering the ongoing human pressure on aquatic communities and explosive road sprawl in many countries, the problem could be more significant at the landscape scale due to the cumulative impacts of road crossings over entire catchments. In this context, further research is essential to monitor the functional characteristics of freshwater ecosystems, as well as to develop future management plans that aim to mitigate these ongoing impacts.

## Methods

### Study sites, environmental variables, sampling and identification

The study area is located in Hungary, Central Europe. The country lies in the Carpathian Basin, where all running waters belong to the river Danube system. The majority of the rivers and streams (ca. 68%) flow through lowland areas (below an altitude of 200 m a.s.l.). We selected nine intersections of roads and small streams (hereafter sites, Suppl. Table 1) which are located outside urban settlements in lowland areas, and where the stream width is less than 10 m. Within each site (e.g. Suppl. Figure 1), we defined a road-crossing section located directly below a bridge, where the length of the sampling reach corresponded to the bridge width (hereafter road crossing section), and two 50-m long sections, one upstream (hereafter upstream section) and one downstream (hereafter downstream section). Upstream and downstream sections were separated by 100 m from the road crossing sections.

The assessment of environmental variables was performed in each stream section before macroinvertebrate sampling. Water physicochemical parameters e.g. temperature, pH, conductivity (μS/cm corrected to 25 °C) and salinity (ppt-parts per thousand) were measured with a Hanna Combo pH/EC/TDS/Temp tester (HI 98129 model). For measuring the dissolved oxygen content (mg/l), we used a Hanna Dissolved Oxygen meter (HI 98193 model). Stream sections were also characterized by four visually estimated environmental variables such as water depth, current velocity, biotic microhabitats and substrate composition. Biotic microhabitats such as emergent macrophytes (*Typha*, *Carex*, *Phragmites*), floating and submerged macrophytes, dead wood and branches and fine roots were quantified using a five-point scale according to their percent cover at a sampling site (1: 1–20%, 2: 20–40%, 3: 40–60%, 4: 60–80%, 5: 80–100%).

Macroinvertebrates were sampled in October (2016), April (2017) and July (2017) using a kick and sweep sampling method. At each site, section (upstream, road crossing, downstream) and sampling date, we took three replicate three-minute samples. Macroinvertebrates were collected in various habitat types, approximately in proportion to their representation of the particular stream section, using D-frame kick net with 500 μm mesh size. Samples were separately preserved in 70% ethanol and identified in the laboratory to the lowest taxonomic unit (usually species)^79,97–105^. We followed the taxonomical nomenclature of the Fauna Europaea Web Service^106^. For more details according to the study sites selection and sampling method see Gál et al.^11^.

### Macroinvertebrate trait data

The database developed by Tachet et al.^107^ was used to define the functional traits of macroinvertebrates. This database contains fuzzy coded traits of macroinvertebrates^108^, where traits are coded by experts^109^. A total of 59 traits from 11 grouping features (describing some general property of species that comprise a group of related traits (see Schmera et al.^110^) were used in this study (Suppl. Table 2): feeding habits, locomotion and substrate preference, respiration, dispersal, maximal potential size, life cycle duration, aquatic stages, potential number of life cycles per year, reproduction, food and resistence forms. The database contained trait data mostly at species and genus level; a total of 21 taxa out of 157^11^ had to be excluded from our analyses because individuals (mostly larvae) could be identified only at a higher (mostly family) taxonomic level (*Curculionidae* sp., *Dryopidae* sp., *Hydraenidae* sp., *Corixidae* sp. larvae, *Notonecitdae* sp. larvae, *Scirtidae* sp., *Limnichidae* sp., *Dryopidae* sp. larvae, *Dytiscidae* sp. larvae, *Haliplidae* sp. larvae, *Hydrophilidae* sp. larvae, *Athericidae* sp., *Ceratopogonidae* sp., *Chironomidae* sp., *Culicidae* sp., *Simuliidae* sp., *Pediciidae* sp., *Erpobdellidae* sp., *Glossiphoniidae* sp., Planaria, Oligochaeta). Trait information for eight additional taxa were not available in the database and thus these taxa were excluded from the analyses (*Argyroneta aquatica*, *Dolomedes fimbriatus*, *Cercyon marinus*, *Cercyon convexiusculus*, *Limnephilus lunatus*, *Limnephilus rhombicus*, *Borysthenia naticina* and *Coelostoma orbiculare)*. Where trait information was only available on genus level, we aggregated the species to the genus level. The trait resolution of database developed by Tachet et al.^107^ is primarily genus-based. We searched also for synonyms according to Fauna Europea^106^. As the measurement of functional diversity (see below) requires that a single community is represented at least by three taxa, some sections had to be omitted from the analyses. Accordingly, Ptychopteridae sp. were omitted from the taxa list, because they were absent in the remaining sections. In total our research used 127 taxa (Suppl. Table 3) and 59 traits from 11 grouping features.

### Measurement of functional diversity

We calculated four measures of functional diversity: functional richness (FRic), functional evenness (FEve), functional divergence (FDiv) and functional dispersion (FDis). FRic is a measure of the overall trait space occupied by the community^32^. FEve describes the evenness of abundance distribution in a functional trait space. Functional divergence (FDiv) refers to the value of species abundances that are present at the fringe of trait space^31^, while FDis measures the dispersion of species in trait space from the centroid^39^.

To compute functional diversity measures, we calculated Gower dissimilarity^111^ using *gowdis* function in *FD* package^112^. Gower dissimilarity tolerates some missing trait values and determines trait distances between taxa based on the original trait dataset. Functional richness, functional evenness, functional dispersion and functional divergence were then calculated using abundance dataset, and the Gower dissimilarity of the trait dataset using *dbFD* function in *FD* package.

For each community we calculated the community-level functional uniqueness and redundancy using the *adiv* pacage and *uniqueness* function^113^, using the measures Ustar (uniqueness) and Rstar (redundancy)^114^. The calculation requires an abundance dataset, and a matrix of dissimilarity between species trait values; we used the Gower dissimilarity. The U* measure is the modified version of the functional uniqueness index by Ricotta et al.^115^. Rao and Gini-Simpson indices are sensitive to replication in species abundance and patterns of difference, although these transformed versions of the Gini-Simpson and Rao indices fulfil the replication principle: replicating N times the composition of a community, the values taken by these transformed indices are multiplied by N^114^. Here the functional uniqueness is the ratio of one minus the Simpson diversity (D) and one minus the Rao quadratic entropy (Q), thus: U* = (1 − D)/(1 − Q). Functional uniqueness measures the decrease in diversity that is obtained by including trait dissimilarities in the calculation of functional diversity^115^. Functional redundancy is the opposite of uniqueness and is defined as species with similar traits within a community that consequently perform the same function in the ecosystem^115^. It was calculated: 1 − U*. If all species in the assemblage are dissimilar from each other then the functional redundancy is zero. In contrast, if all species are functionally identical, then the redundancy is one. Analyses were done using R (R version 4.1.1.) with the *FD* package^112^.

### Statistical analyses

We used linear models to examine whether the functional diversity measures (richness, evenness, dispersion and divergence) and functional uniqueness were influenced by the stream section (i.e. upstream, road crossing and downstream), study sites and seasons. We selected the best-fit models using an information theoretic approach based on the Akaike Information Criterion corrected for the number of cases and estimated parameters (AICc), and Akaike weights^116^. Delta AICc is the difference in the fit between a particular model considered and that of the best fit model. Models with delta AICc < 10 are only considered. AIC weight was calculated among all possible pairs and indicates the probability of the model. If the ANOVA table of the best fit model revealed significant differences, then a Tukey test^117^ was used for multiple comparison. For this calculation was We used *multcomp*^118^ and *MuMIn*^119^ packages.

### Trait response to the road crossing structure

RLQ analysis was used to assess global relationships between the biological trait composition of the macroinvertebrate assemblages and the environmental variables^120,121^. The data used in this analysis consisted of three matrixes: R matrix (environmental variables), Q matrix (macroinvertebrate traits) and L matrix (abundance). In the first step we used three separate ordination methods on each matrix. Correspondence analysis was performed on the abundance table, Principal Components Analysis on the environmental variable table and Hill- Smith analysis on the functional trait table. The next step in the RLQ analysis combines the three ordinations via co-inertia techniques to identify the primary relationships between environmental characteristics and functional traits which are mediated by species abundances at sampling sites^122^.

A multivariate statistic was used to test the significance of the global association between the three tables. We performed a Monte Carlo test (9999 random permutations)^120,121^ and used two models. The first model tested the null hypothesis that the distribution of macroinvertebrate taxa was not influenced by the environmental variables (model 2). The second model tested the null hypothesis that traits did not influence the composition of species found in a stream section with certain environmental conditions (model 4)^123^. For this calculation we used the *ade4* package^124^.

The fourth-corner analysis allowed us to test multiple associations between single traits and environmental variables, which can reveal more detailed information on trait-environment associations. The data used in this analysis consisted of three matrices: R matrix (environmental variables), Q matrix (macroinvertebrate traits) and L matrix (abundance) to detect univariate correlations between each combination of trait-environment variables. The analysis used two sequential permutation models as recommended by Dray and Legendre^121^ and ter Braak et al.^125^. The first model (model 2) tested the null hypothesis that there is no link between the environmental variables and species abundances (R and L tables). If the null hypothesis was rejected, then the second model (model 4) was applied to test the null hypothesis that there is no link between species composition of samples and functional trait characteristics (L and Q tables). The tests were carried out using 999 permutations and used the Pearson product- moment correlation coefficient (r) to obtain the strength and direction (positive or negative) of the association between the environmental variables and traits. For the fourth corner analysis, we used function *fourthcorner* included in the package *ade4*^124^ implemented in the R software (R version 4.1.1.).

Finally, fourth-corner tests were directly applied to the multivariate RLQ analysis. Each method has a drawback if implemented separately. RLQ analysis alone does not provide a significant test between trait and environmental relationships, while fourth- corner analysis does not consider the covariation among traits and environmental variables. Dray et al.^123^ proposed a framework for joint use of these two complementary methods to describe multivariate patterns and to test the significant bivariate associations. Pearson’s correlation tests were used to quantify the relationship between RLQ axes and individual traits and/or environmental variables.

### Supplementary Information

Supplementary Information.

## Data Availability

The datasets that support the findings of this study are available on request to the corresponding author.
