# Supplementary material for: Road crossings change functional diversity and trait composition of stream-dwelling macroinvertebrate assemblages
Source: Sci Rep. 2023 Nov 24;13:20698. doi: 10.1038/s41598-023-47975-z (PMC10674018; doi:10.1038/s41598-023-47975-z)
Supplement: Supplementary file 1 — Supplementary Information. [file 41598_2023_47975_MOESM1_ESM.docx]

Supplementary information for

**Road crossings change functional diversity and trait composition of stream-dwelling macroinvertebrate assemblages**

Blanka Gál^1,2,^*, András Weiperth^3,4^, János Farkas^4^, Dénes Schmera^1,2^

^1^Balaton Limnological Research Institute, Klebelsberg K. u. 3, H-8237 Tihany, Hungary

^2^National Laboratory for Water Science and Water Security, Balaton Limnological Research Institute, Klebelsberg K. u. 3, H-8237 Tihany, Hungary

^3^Department of Freshwater Fish Ecology, Institute of Aquaculture and Environmental Safety, Hungarian University of Agriculture and Life Sciences, Páter Károly u. 1, H-2103 Gödöllő, Hungary

^4^Eötvös Loránd University, Department of Systematic Zoology and Ecology, Institute of Biology, ELTE Eötvös Loránd University, Pázmány Péter sétány 1/C, H- 1117 Budapest, Hungary

*Correspondence: gal.blanka@blki.hu

Suppl. Table 1: List of studied sites

| Code | Name | GPS Coordinates |
| --- | --- | --- |
| Site 1 | M6 motorway | 47°13'17.3"N 18°49'10.5"E |
| Site 2 | M7 motorway | 47°18'08.5"N 18°44'31.3"E |
| Site 3 | M3 motorway | 47°41'01.4"N 19°39'55.9"E |
| Site 4 | M85 expressway | 47°33'24.2"N 17°14'48.5"E |
| Site 5 | M86 expressway | 47°37'46.8"N 17°19'40.5"E |
| Site 6 | bike path | 47°43'42.9"N 19°08'02.7"E |
| Site 7 | main road 2 | 47°43'42.2"N 19°08'09.6"E |
| Site 8 | train bridge | 47°43'26.9"N 19°09'15.5"E |
| Site 9 | M2 motorway | 47°43'26.8"N 19°09'25.4"E |

Suppl. Table 2: List of traits

| **Grouping feature** | **Trait** |  |
| --- | --- | --- |
|  |  | |
| **feeding habits** | deposit feeder | |
|  | shredder | |
|  | scraper | |
|  | filter-feeder | |
|  | piercer (plants or animals) | |
|  | predator (carver/engulfer/swallower) | |
|  | parasite | |
| **locomotion and substrate relation** | flier | |
|  | surface swimmer | |
|  | full water swimmer | |
|  | crawler | |
|  | burrower (epibenthic) | |
|  | interstitial (endobenthic) | |
|  | temporarily attached | |
| **respiration** | tegument | |
|  | gill | |
|  | plastron | |
|  | spiracle (aerial) | |
| **dispersal** | aquatic passive | |
|  | aquatic active | |
|  | aerial passive | |
|  | aerial active | |
| **maximal potential size** | <= 0.25 cm | |
|  | > 0.25 - 0.5 cm | |
|  | > 0.5 - 1 cm | |
|  | > 1 - 2 cm | |
|  | > 2 - 4 cm | |
|  | > 4 - 8 cm | |
|  | > 8 cm | |
| **life cycle duration** | <= 1 year | |
|  | > 1 year | |
| **aquatic stages** | egg | |
|  | larva | |
|  | nymph/pupa | |
|  | adult | |
| **potential number of cycles per year** | life cycle lasts at least two years (semivoltine) | |
|  | one generation per year (monovoltine) | |
|  | more than two generations per year (polyvoltine) | |
| **reproduction** | ovoviviparity | |
|  | isolated eggs, free | |
|  | isolated eggs, cemented | |
|  | clutches, cemented or fixed | |
|  | clutches, free | |
|  | clutches, in vegetation | |
|  | clutches, terrestrial | |
| **food** | fine sediment + microorganisms | |
|  | fine detritus (≤ 1mm) | |
|  | dead plants (> 1mm) | |
|  | living microphytes | |
|  | living macrophytes | |
|  | dead animals (> 1mm) | |
|  | living microinvertebrates | |
|  | living macroinvertebrates | |
|  | vertebrates | |
| **resistance forms** | eggs, gemmula, statoblasts | |
|  | cocoons | |
|  | housings against desiccation | |
|  | diapause or dormancy | |
|  | none | |

Suppl. Table 3: List of 127 macroinvertebrate taxa

| **Group** | **Taxon** |
| --- | --- |
| **Hirudinea** | Haemopidae sp. |
|  | Piscicolidae sp. |
| **Amphipoda** | *Dikerogammarus bispinosus Martynov, 1925* |
|  | *Echinogammarus ischnus (Stebbing, 1899)* |
|  | *Gammarus fossarum Koch, in Panzer, 1835* |
|  | *Gammarus roeseli* Gervais, 1835 |
| **Isopoda** | *Asellus aquaticus* (Linnaeus, 1758) |
| **Mysida** | *Limnomysis benedeni* Czerniavsky, 1882 |
| **Coleoptera** | *Agabus (Agabus) undulatus* (Schrank, 1776) |
|  | *Ilybius fuliginosus* (Fabricius, 1792) |
|  | *Platambus maculatus* (Linnaeus, 1758) |
|  | *Colymbetes fuscus* (Linnaeus, 1758) |
|  | *Rhantus (Rhantus) suturalis* (MacLeay, 1825) |
|  | *Cybister (Scaphinectes) lateralimarginalis* (De Geer, 1774) |
|  | *Hydaticus (Hydaticus) transversalis* (Pontoppidan, 1763) |
|  | *Hydroglyphus geminus* (Fabricius, 1792) |
|  | *Graptodytes pictus* (Fabricius, 1787) |
|  | *Hydroporus angustatus* Sturm, 1835 |
|  | *Porhydrus obliquesignatus* (Bielz, 1852) |
|  | *Hygrotus (Coelambus) impressopunctatus* (Schaller, 1783) |
|  | *Hygrotus (Hygrotus) inaequalis* (Fabricius, 1776) |
|  | *Hygrotus (Hygrotus) versicolor* (Schaller, 1783) |
|  | *Hyphydrus ovatus* (Linnaeus, 1761) |
|  | *Laccophilus hyalinus* (De Geer, 1774) |
|  | *Laccophilus minutus* (Linnaeus, 1758) |
|  | *Laccophilus poecilus* Klug, 1834 |
|  | *Haliplus (Haliplus) fluviatilis* Aube, 1836 |
|  | *Haliplus (Haliplus) heydeni* Wehncke, 1875 |
|  | *Haliplus (Haliplus) immaculatu*s Gerhardt, 1877 |
|  | *Haliplus (Neohaliplus) lineatocollis* (Marsham, 1802) |
|  | *Haliplus* sp. |
|  | *Peltodytes caesus* (Duftschmid, 1805) |
|  | *Noterus clavicornis* (De Geer, 1774) |
|  | *Noterus crassicornis* (O. F. Muller, 1776) |
|  | *Helophorus (Rhopalohelophorus) minutus* Fabricius, 1775 |
|  | *Helophorus (Rhopalohelophorus) granularis* (Linnaeus, 1761) |
|  | *Helophorus* sp. larvae |
|  | *Anacaena limbata* (Fabricius, 1792) |
|  | *Berosus (Berosus) luridus* (Linnaeus, 1761) |
|  | *Hydrochara caraboides* (Linnaeus, 1758) |
|  | *Hydrophilus (Hydrophilus) aterrimus* Eschscholtz, 1822 |
|  | *Limnoxenus niger* (Gmelin, 1790) |
| **Diptera** | Ephydridae sp. |
|  | Muscidae sp. |
|  | Stratiomyidae sp. |
|  | Tabanidae sp. |
|  | Tipulidae sp. |
| **Ephemeroptera** | *Baetis rhodani* (Pictet, 1843) |
|  | *Baetis* sp. |
|  | *Centroptilum luteolum* (Muller, 1776) |
|  | *Cloeon dipterum* (Linnaeus, 1761) |
|  | *Caenis luctuosa* (Burmeister, 1839) |
|  | *Caenis robusta* Eaton, 1884 |
|  | *Ephemera vulgata* Linnaeus, 1758 |
|  | *Potamanthus luteus* (Linnaeus, 1767) |
|  | *Heptagenia flava* Rostock, 1878 |
| **Hemiptera** | *Corixa punctata* (Illiger, 1807) |
|  | *Hesperocorixa linnaei* (Fieber, 1848) |
|  | *Hesperocorixa sahlbergi* (Fieber, 1848) |
|  | *Sigara (Subsigara) falleni* (Fieber, 1848) |
|  | *Sigara (Vermicorixa) lateralis* (Leach, 1817) |
|  | *Sigara (Sigara) striata* (Linnaeus, 1758) |
|  | *Micronecta (Dichaetonecta) scholtzi* (Fieber, 1860) |
|  | *Micronecta (Micronecta) poweri* (Douglas & Scott, 1869) |
|  | Gerridae sp. |
|  | *Microvelia* sp. |
|  | *Hydrometra gracilenta* Horvath, 1899 |
|  | *Hydrometra stagnorum* (Linnaeus, 1758) |
|  | *Ilyocoris cimicoides* (Linnaeus, 1758) |
|  | *Nepa cinerea* Linnaeus, 1758 |
|  | *Notonecta (Notonecta) glauca* Linnaeus, 1758 |
|  | *Notonecta (Notonecta) viridis* Delcourt, 1909 |
|  | *Plea minutissima* Leach, 1817 |
| **Odonata** | *Aeshna affinis* Vander Linden, 1820 |
|  | *Aeshna cyanea* (Muller, 1764) |
|  | *Aeshna isoceles* (Muller, 1767) |
|  | *Aeshna mixta* Latreille, 1805 |
|  | *Gomphus vulgatissimus* (Linnaeus, 1758) |
|  | *Cordulia aenea* (Linnaeus, 1758) |
|  | *Libellula depressa* Linnaeus, 1758 |
|  | *Orthetrum brunneum* (Fonscolombe, 1837) |
|  | *Orthetrum coerulescens* (Fabricius, 1798) |
|  | *Calopteryx splendens* (Harris, 1782) |
|  | *Calopteryx virgo* (Linnaeus, 1758) |
|  | *Coenagrion ornatum* (Selys, 1850) |
|  | *Coenagrion puella* (Linnaeus, 1758) |
|  | *Coenagrion pulchellum* (Vander Linden, 1825) |
|  | *Enallagma cyathigerum* (Charpentier, 1840) |
|  | *Ischnura elegans* (Vander Linden, 1820) |
|  | *Ischnura pumilio* (Charpentier, 1825) |
|  | *Platycnemis pennipes* (Pallas, 1771) |
| **Trichoptera** | *Hydropsyche angustipennis* (Curtis, 1834) |
|  | *Hydropsyche modesta* Navàs, 1925 |
|  | *Athripsodes bilineatus* (Linnaeus, 1758) |
|  | *Athripsodes cinereus* (Curtis, 1834) |
|  | *Oecetis lacustris* (Pictet, 1834) |
|  | *Oecetis ochracea* (Curtis, 1825) |
|  | *Lepidostoma hirtum* (Fabricius, 1775) |
|  | *Hydroptila* sp. |
| **Veneroidea** | *Pisidium amnicum* (O. F. Muller, 1774) |
|  | *Pisidium* sp. |
|  | *Sphaerium corneum* (Linnaeus, 1758) |
| **Unionoida** | *Unio pictorum* (Linnaeus, 1758) |
| **Architaenioglossa** | *Viviparus contectus* (Millet, 1813) |
| **Neotaenioglossa** | *Bithynia tentaculata* (Linnaeus, 1758) |
|  | *Bithynia troschelii* (Paasch, 1842) |
|  | *Potamopyrgus antipodarum* (J.E. Gray, 1843) |
| **Ectobranchia** | *Valvata (Cincinna) piscinalis* (O.F. Muller, 1774) |
|  | *Valvata (Tropidina) macrostoma* Morch, 1864 |
|  | *Valvata (Valvata) cristata* O.F. Muller, 1774 |
| **Pulmonata** | *Acroloxus lacustris* (Linnaeus, 1758) |
|  | *Galba (Galba) truncatula* (O.F. Muller, 1774) |
|  | *Lymnaea stagnalis* (Linnaeus, 1758) |
|  | *Radix auricularia* (Linnaeus, 1758) |
|  | *Radix labiata* (Rossmassler, 1835) |
|  | *Stagnicola turricula* (Held, 1836) |
|  | *Haitia acuta* (Draparnaud, 1805) |
|  | *Physa fontinalis* (Linnaeus, 1758) |
|  | *Planorbarius corneus* (Linnaeus, 1758) |
|  | *Anisus (Anisus) septemgyratus* (Rossmassler, 1835) |
|  | *Anisus (Disculifer) vortex* (Linnaeus, 1758) |
|  | *Anisus (Disculifer) vorticulus* (Troschel, 1834) |
|  | *Gyraulus (Armiger) crista* (Linnaeus, 1758) |
|  | *Gyraulus (Gyraulus) albus* (O.F. Muller, 1774) |
|  | *Hippeutis complanatus* (Linnaeus, 1758) |
|  | *Planorbis (Planorbis) planorbi*s (Linnaeus, 1758) |
|  | *Segmentina nitida* (O.F. Muller, 1774) |


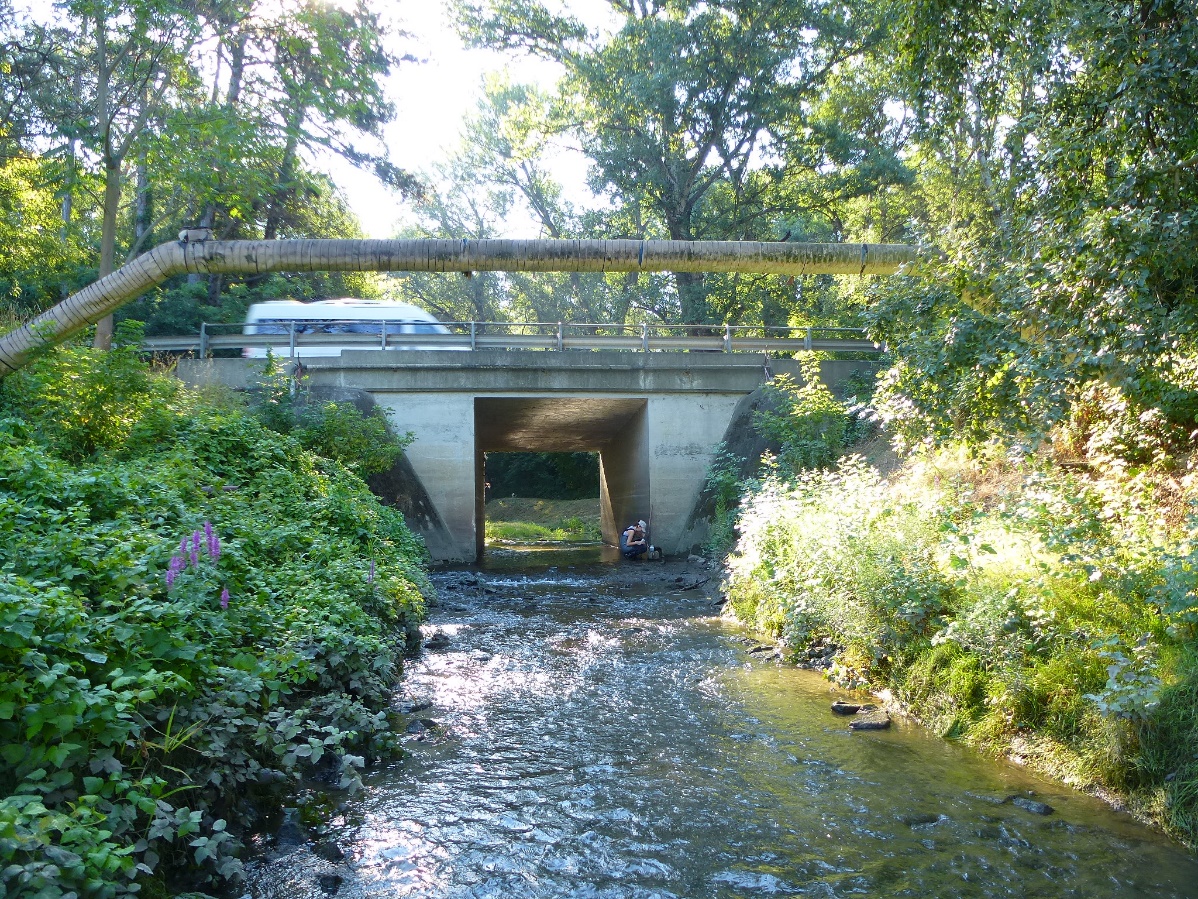


Suppl. Fig. 1: Road crossing of Sződrákos stream and main road 2 in Hungary


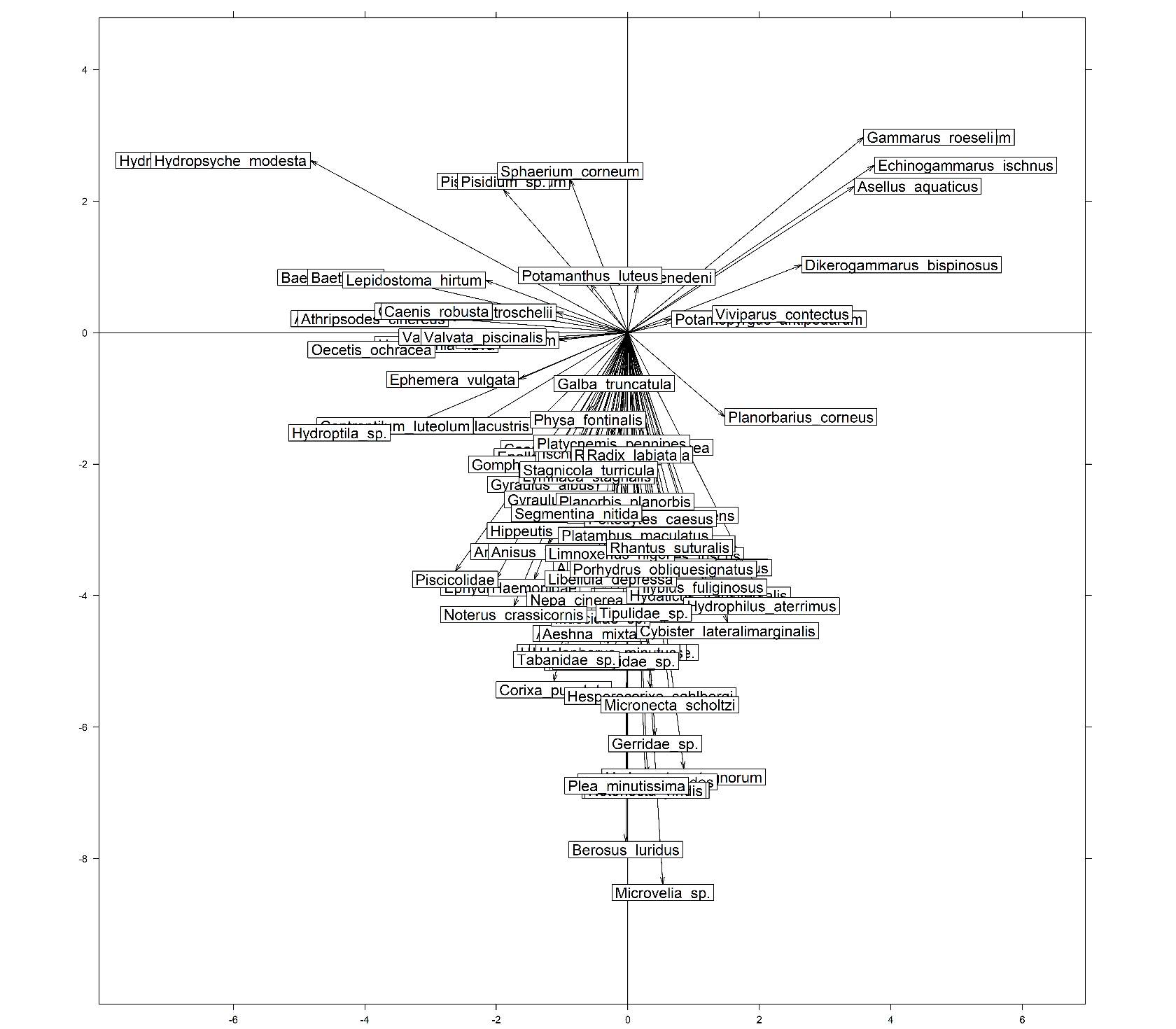


Suppl. Fig. 2: First two axes of RLQ ordination plots showing the distribution of taxa by their traits


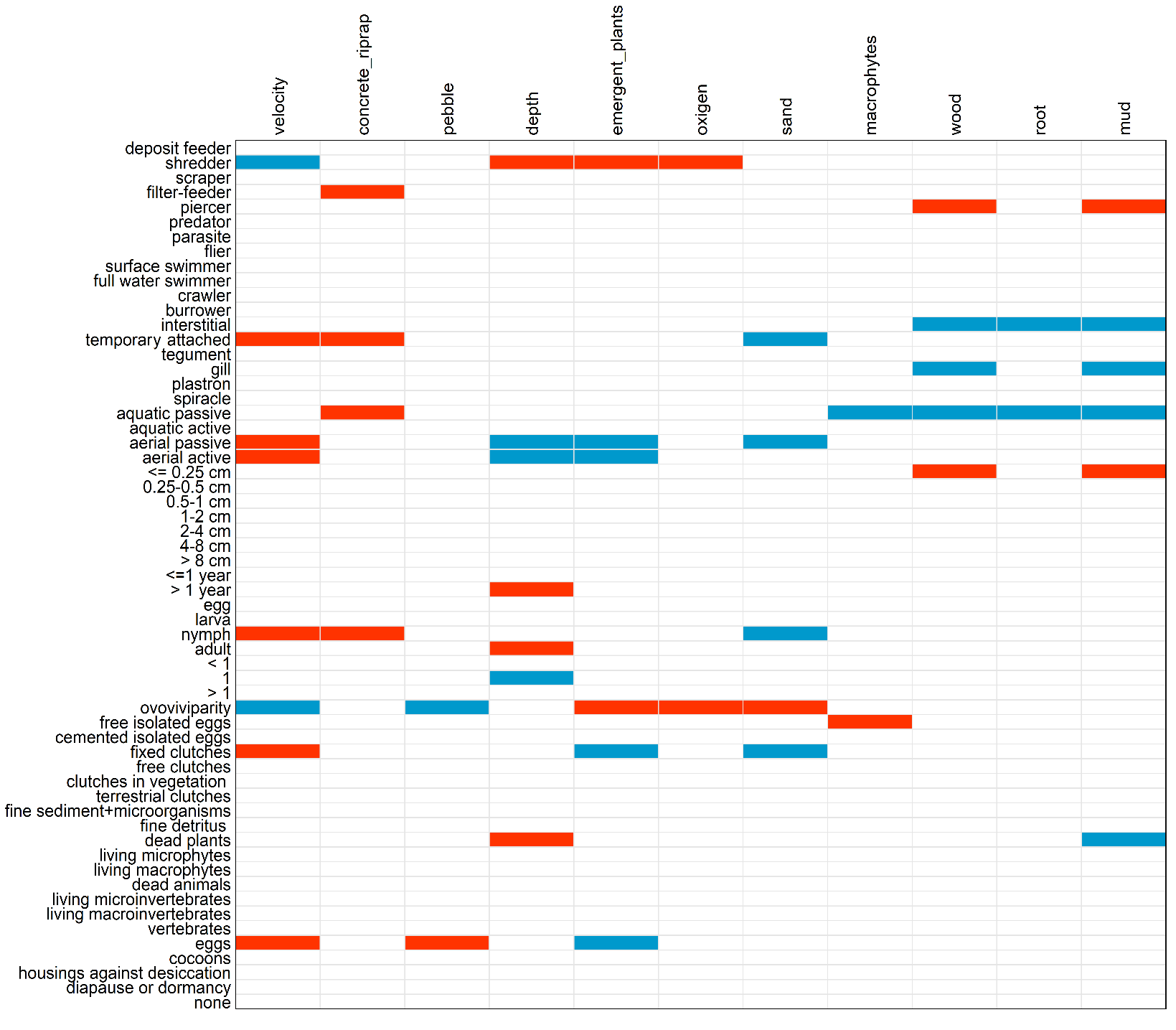


Suppl. Fig. 3: Results of a fourth-corner analyses that tested the associations between functional traits and environmental variables. Coloured cells show significant associations at the P < 0.05 level. Red cells= positive associations; blue cells = negative associations; white cells= non-significant associations
